# Left atrial strain parameters for predicting new-onset atrial fibrillation: a systematic review and meta-analysis

**DOI:** 10.3389/fcvm.2026.1745597

**Published:** 2026-06-03

**Authors:** Haijian Yang, Youfu He, Lei Peng, Deqi Wang, Jun Yang, Linfeng Yin

**Affiliations:** 1 Department of Cardiology, Linping Hospital of Integrated Traditional Chinese and Western Medicine, Hangzhou, Zhejiang, China; 2 Department of Cardiology, Guizhou Provincial People’s Hospital, Guiyang, Guizhou, China; 3 Department of Interventional Cardiology, ZaoZhuang Municipal Hospital, Zaozhuang, Shandong, China; 4 Ultrasound Department, Hangzhou Linping District Maternal & Child Health Care Hospital, Hangzhou, Zhejiang, China

**Keywords:** left atrial strain, meta-analysis, new-onset atrial fibrillation, prediction, systematic review

## Abstract

**Background:**

Early identification of new-onset atrial fibrillation (NOAF) is crucial for preventing related complications. Left atrial strain parameters (LASPs), evaluated by speckle tracking echocardiography, are considered promising markers reflecting atrial cardiomyopathy and functional abnormalities. Therefore, this study aimed to quantitatively evaluate the predictive value of LASPs for NOAF through a systematic review and meta-analysis.

**Methods:**

The Cochrane Library, Embase, PubMed, and Web of Science were retrieved up to July 30, 2025, for cohort, case-control, and cross-sectional investigations evaluating the correlation of peak atrial longitudinal strain (PALS), left atrial reservoir strain (LASr), left atrial conduit strain (LAScd), left atrial systolic strain (LASct), and peak atrial contraction strain (PACS) with the risk of NOAF. Random-effects models were employed to pool risk ratios (RR) and diagnostic performance metrics. Sensitivity analyses and Egger's test were applied to verify the stability of prognostic outcomes and publication bias, respectively. Subgroup and meta-regression analyses were conducted for multiple confounders. A summary receiver operating characteristic (SROC) curve was plotted, and the area under the curve (AUC) was computed.

**Results:**

Thirty-nine studies involving 20,681 subjects were incorporated. Meta-analyses revealed that low levels of PALS [RR (95% CI): 3.95 (1.57–9.92)], LASr [3.73 (2.34–5.96)], LAScd [2.26 (1.22–4.19)], and LASct [6.54 (2.25–19.0)] as dichotomous variables were associated with a higher risk of NOAF. As continuous variables, a one-unit decrease in PALS, PACS, LASr, LAScd, and LASct was independently associated with an elevated risk of NOAF (all *P* < 0.05). SROC analysis indicated that the pooled AUCs for PALS, LASr, and LASct were 0.84, 0.84, and 0.78, respectively, suggesting good diagnostic performance for these parameters.

**Conclusion:**

LASPs exhibit significant diagnostic value for NOAF. They can be utilized in clinical practice to evaluate and predict the risk of NOAF.

**Systematic Review Registration:**

https://www.crd.york.ac.uk/PROSPERO/view/CRD420251044556.

## 1 Introduction

Atrial fibrillation (AF), a pervasive and persistent cardiac arrhythmia globally, exhibits increasing prevalence and burden with an aging population. A report from the Framingham Heart Study revealed a 2-fold escalation in the prevalence of AF over the past half-century (1, 2). Individuals diagnosed with new-onset atrial fibrillation (NOAF) endure symptoms like palpitations and fatigue and face substantially elevated risks of stroke (3), heart failure (4, 5), cognitive decline (6), and adverse cardiovascular events (7). Current guidelines advocate for opportunistic and systematic screening for AF diagnosis (8). Nonetheless, a considerable proportion of patients experience atrial remodeling during asymptomatic phases, leading to detection only after stroke or decompensated heart failure. Consequently, identifying imaging biomarkers capable of capturing functional anomalies before structural changes occur holds significant importance.

Atrial cardiomyopathy is recognized as a crucial underlying substrate for the development of AF, involving structural and functional alterations in atrial cardiomyocytes (9, 10). Extensive prior research indicates that atrial cardiomyopathy contributes to the pathophysiology of AF and serves as a primary driver for its onset and recurrence (9, 11). Investigations into atrial cardiomyopathy have progressively illuminated the roles of atrial remodeling, atrial dilation, and electrophysiological abnormalities in the pathogenesis of AF (11, 12). With ongoing research, atrial cardiomyopathy is now considered a key predictive marker for AF. Meanwhile, morphological and functional atrial changes offer novel perspectives for forecasting the early manifestation of AF (13). Understanding the association of atrial cardiomyopathy with AF provides a fundamental basis for exploring correlations of atrial indices with AF in this meta-analysis.

In recent years, speckle tracking echocardiography (STE) has emerged as a novel echocardiographic technique demonstrating considerable potential in assessing atrial function (14). STE quantifies myocardial strain and strain rate, providing dynamic cardiac functional information clinically. It holds particular diagnostic value concerning parameters such as atrial strain, systolic strain, and reservoir strain (15). Numerous studies have established the efficacy of STE in evaluating atrial remodeling and functional changes, offering valuable insights for early prediction of AF (16). Nevertheless, despite accumulated clinical experience with STE for the evaluation of atrial function, its application in identifying early AF remains constrained. Precisely interpreting these indices across diverse populations and clinical contexts presents a current research challenge.

This meta-analysis comprehensively integrates the association of left atrial strain parameters (LASPs) (peak atrial longitudinal strain [PALS], peak atrial contraction strain [PACS], left atrial reservoir strain [LASr], left atrial conduit strain [LAScd], left atrial systolic strain [LASct]) with NOAF. It further explores their potential in diagnosing early AF. The aim is to elucidate the applicability of atrial strain parameters across different populations, thereby providing a theoretical foundation for the prediction and intervention of early AF.

## 2 Materials and methods

### 2.1 Protocol registration

The present meta-analysis was registered with the International Prospective Register of Systematic Reviews (PROSPERO; CRD420251044556). No protocol deviations have occurred in this study. The research adhered to the Preferred Reporting Items for Systematic Reviews and Meta-Analyses (PRISMA) guidelines (17).

### 2.2 Literature search

The Cochrane Library, Web of Science, PubMed, and Embase were retrieved up to July 30, 2025. Identified keywords comprised PALS, PACS, LASr, LAScd, LASct, and AF. Medical subject headings and free-text terms were combined using Boolean operators (AND, OR, NOT). Details are illustrated in Supplementary Table S1. Additionally, reverse citation tracking was performed on systematic and narrative reviews identified, as well as on all eligible trials.

### 2.3 Study selection

Articles obtained from the initial search were screened at the title and abstract level by 2 independent reviewers (HY and LY). Full-text articles were further evaluated to determine eligibility for the final analysis. Discrepancies were resolved through consensus with an expert (LP). Inclusion criteria comprised: (i) study participants without a known history of paroxysmal or persistent AF; (ii) study designs including cohort, case-control, and cross-sectional studies; (iii) testing data encompassing PALS, PACS, LASr, LAScd, and LASct; (iv) extracted outcomes including the risk ratio (RR) of left atrial strain for NOAF with its 95% confidence interval (CI), sensitivity, specificity, and the area under the summary receiver operating characteristic (SROC) curve (sAUC); and (v) publications in English. Exclusion criteria incorporated: (i) case reports, reviews, letters, conference abstracts, and commentaries; (ii) animal experimental studies; (iii) studies with duplicated or overlapping data; (iv) inability to access full-text articles; and (v) non-English literature.

### 2.4 Information collection

Two independent researchers (HY and YH) evaluated eligible studies and collected information. Extracted information encompassed first author, publication year, country, study design, study population, sample size, age, sex, hypertension, diabetes mellitus (DM), coronary artery disease, LASPs, follow-up duration, and AF diagnostic criteria. When direct data for true positives, false positives, false negatives, and true negatives were unavailable, these values were computed by the 2 researchers based on sensitivity and specificity. Discrepancies were resolved through consultation with a third researcher.

### 2.5 Quality evaluation

For diagnostic accuracy studies, the quality was evaluated employing the Quality Assessment of Diagnostic Accuracy Studies 2 (QUADAS-2) tool in RevMan 5.3 (18). This involved evaluating the risk of bias and applicability for each eligible publication across 4 key domains: patient selection, index tests, reference standard, and flow and timing. For investigations involving only correlation analyses, the Newcastle-Ottawa Scale (NOS) was employed to appraise the quality (19). The NOS evaluates study quality based on selection (4 points), comparability (2 points), and outcome and follow-up ascertainment (3 points). Studies with an NOS score (range: 0 to 9) greater than 6 were considered high quality. Two reviewers (HY and DW) independently evaluated the risk of bias for each eligible study, with any discrepancies resolved by consensus.

### 2.6 Statistical analysis

Statistical computations were conducted using Stata 15.0 and Meta-Disc 1.4. Initially, heterogeneity among the eligible studies was tested using the I² statistic to determine the presence and magnitude of heterogeneity. If *I*² ≤ 50% and *P* > 0.1, indicating low heterogeneity, a fixed-effects model was utilized for meta-analysis; otherwise, a random-effects model was employed. RRs and their 95% CIs were pooled. Subgroup analyses and meta-regression were conducted to identify sources of heterogeneity (20). Sensitivity analyses were carried out by sequentially omitting individual studies to evaluate the robustness of the findings. Publication bias was evaluated via Egger's test and funnel plots. For diagnostic data, Meta-Disc 1.4 was adopted to test for threshold effects, calculating the Spearman correlation coefficient and *P*-value between the logarithm of sensitivity and the logarithm of (1-specificity). *P* > 0.05 indicated the absence of a threshold effect. Based on heterogeneity, appropriate effect models were selected for meta-analyses to calculate diagnostic test indices, including sensitivity, specificity, diagnostic odds ratio (DOR), likelihood ratios, and sAUC. Fagan nomograms were applied to illustrate the relationship between pre-test (clinician's evaluation based on subject history, signs, and personal experience) and post-test probabilities (prevalence after diagnostic testing). Deeks' funnel plot assessed publication bias. All statistical tests were two-sided, with *P* < 0.05 indicating statistical significance.

## 3 Results

### 3.1 Characteristics of eligible studies

**Figure 1:**
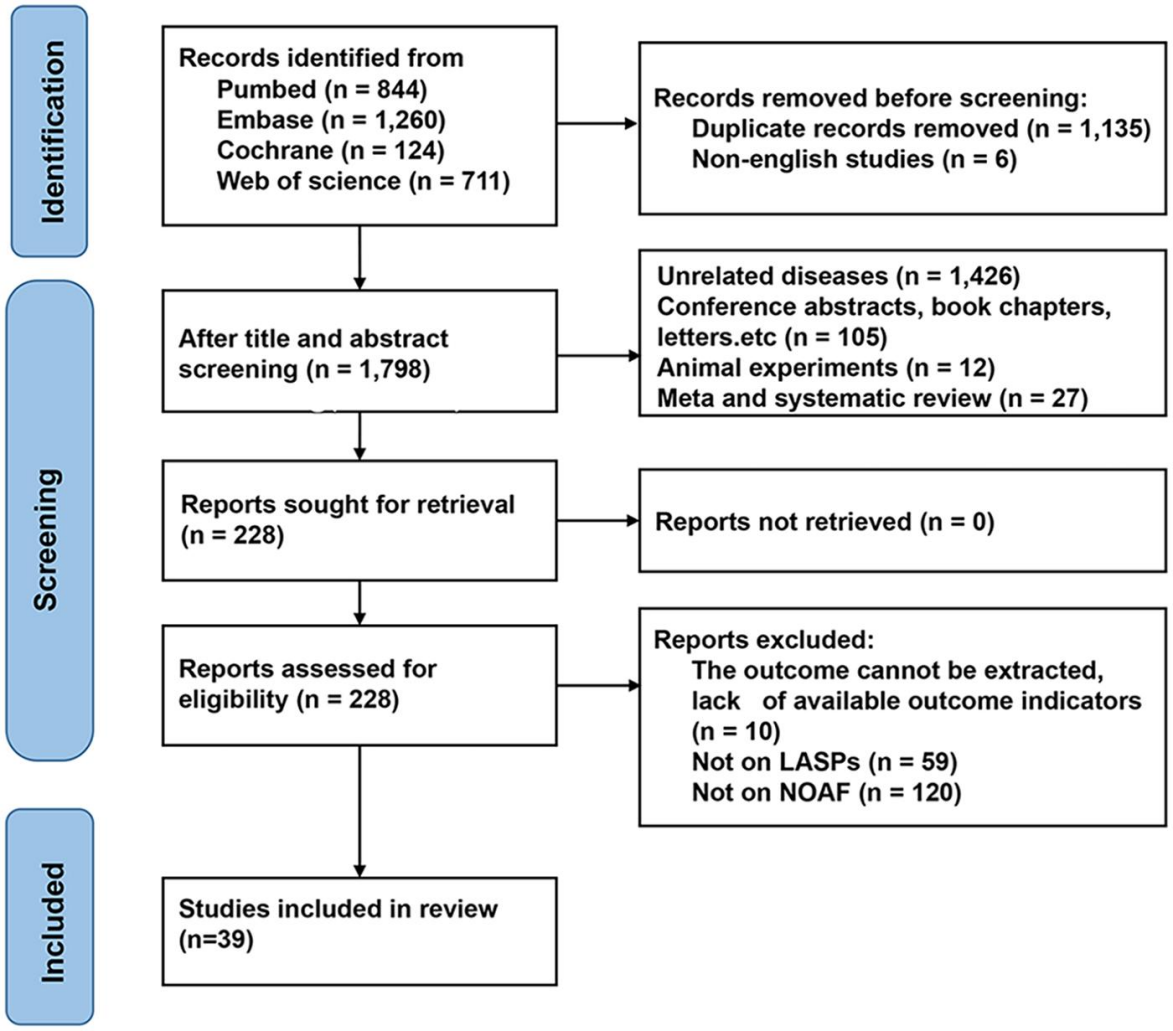
Literature screening flowchart.

An initial search of the 4 major databases yielded 2,939 articles. After removing 1,135 duplicates, 6 non-English articles, and 12 animal experimental studies, 1,558 studies were excluded by reviewing the title and abstract. Subsequently, 189 of the remaining 228 studies were excluded through a full-text review. The main reasons for exclusion were irrelevance to NOAF (*n* = 120), lack of association with LASPs (*n* = 59), and insufficient effective data (*n* = 10). Ultimately, this meta-analysis incorporated 39 studies (21–59) (Figure 1). The sample sizes of these eligible studies ranged from 56 to 5,050 individuals, for a total of 20,681 subjects. The mean age was 63.83 ± 15.52 years. Details are presented in Table 1. The mea*n* ± standard deviation of LASPs in patients with NOAF is presented in Supplementary Table S2.

**Table 1:** Basic characteristics of the eligible studies.

Author	Year	Country	Design	N	Age	Males (%)	Surgery (%)	Hypertension	Diabetes	Coronary artery disease	LA strain	Follow up	AF diagnosis	Measuring technique	Software
Abdelrazek	2021	Egypt	Case control	89	56.16 ± 7.54	65.17	100	65.17	51.69	100.00	PALS	NA	ECG	2D-STE	QLAB (Philips)
Alhakak	2022	Denmark	Retrospective cohort study	400	56.00 ± 16.00	41.00	NA	36.00	8.00	NA	PALS	17 years	NA	2D-STE	EchoPAC (GE)
Arnăutu	2022	Romania	Retrospective cohort study	190	64.00 ± 7.00	64.00	NA	70.00	17.00	NA	LASr, LAScd	NA	ECG/Holter	2D-STE	EchoPAC (GE)
Beyls	2024	France	Prospective cohort study	175	60.18 ± 12.97	74.86	0.57	50.86	15.43	100.00	LASr, LAScd, LASct	6 months	ECG	2D-STE	Auto-Strain QLAB (Philips)
Cameli	2014	Italy	Prospective cohort study	76	67.49 ± 11.84	68.00	100	65.80	15.80	0.00	PALS	NA	ECG	2D-STE	EchoPAC (GE)
Cauwenberghs	2020	Belgium	Prospective cohort study	1,306	50.7. ± 15.7.	48.40	0	42.40	4.20	NA	LASr	8.4 ± 3.6year	NA	2D-STE	Q-analysis (GE)
Dalos	2022	Austria	Prospective cohort study	124	71.21 ± 8.55	67.70	100	79.80	26.60	51.60	PALS	3.76 ± 2.25year	ECG	2D-STE	EchoPAC (GE)
Deferm	2021	United States	Retrospective cohort study	191	65.00 ± 14.00	52.88	NA	60.73	18.32	NA	PALS	26.05 ± 24.65months	Mobile Cardiac Outpatient Telemetry	2D-STE	TomTec
Granchietti	2025	Italy	Prospective cohort study	100	67.70 ± 8.80	89.00	100	83.00	42.00	100.00	LASr, LASct	13.81 ± 6.12days	ECG	2D-STE	Canon
Hauser	2022	Denmark	Prospective cohort study	3,590	55.30 ± 17.40	43.20	NA	48.50	4.30	NA	PALS, PACS, LAScd	5.3year	ICD-10	2D-STE	EchoPAC (GE)
Inciardi	2024	United States	Prospective cohort study	5050	75.00 ± 5.00	41.00	NA	71.30	29.90	11.40	LASr, LAScd, LASct	7years	ECG/ICD	2D-STE	TomTec/QLAB
Jasic-Szpak	2021	Poland	Prospective cohort study	170	65.00 ± 8.00	27.10	NA	91.20	39.40	0.00	PALS, PACS, LAScd	49months	ECG/Holter	2D-STE	EchoPAC (GE)
Kawakami	2019	Australia	Case control	70	70.00 ± 4.00	57.10	NA	75.70	54.30	NA	LASr	15months	ECG	2D-STE	TomTec
Kawakami	2020	Australia	Retrospective cohort study	531	67.00 ± 16.35	56.00	NA	69.00	19.00	12.00	LASr, LAScd	36.35 ± 26.02	ECG/Holter	2D-STE	TomTec
Kislitsina	2022	United States	Retrospective cohort study	211	64.60 ± 9.60	82.00	100	NA	NA	100.00	LASr	NA	NA	2D-STE	TOMTEC
Kosmala	2015	Australia, New Zealand, United Kingdom	Prospective cohort study	146	73.00 ± 10.00	67.80	100	57.50	21.90	26.00	LASct	2years	ECG	2D-STE	TomTec
Kusunose	2021	Japan	Prospective cohort study	121	75.00 ± 13.00	59.00	NA	59.00	24.00	24.00	LASr, LAScd	16.24 ± 8.25days	ECG	2D-STE	Echolnight (Epsilon)
Lohrmann	2020	United States	Retrospective cohort study	91	58.00 ± 9.00	58.24	100	40.66	5.49	1.10	LASr, LASct	NA	ECG	2D-STE	TomTec
Malagoli	2019	Italy	Prospective cohort study	286	67.00 ± 11.00	81.12	NA	71.33	23.08	64.34	PALS	48 ± 11months	NA	2D-STE	EchoPAC (GE)
Mannina	2023	United States	Prospective cohort study	824	71.10 ± 9.20	38.00	NA	79.00	29.70	5.70	PALS, LASct	10.9 ± 3.7years	ECG	2D-STE	TomTec
Nagi	2023	Egypt	Prospective cohort study	129	58.80 ± 8.90	86.00	100	76.00	73.60	100.00	LASr, LAScd, LASct	9.7days	ECG	2D-STE	NA
Olsen	2020	Denmark	Prospective cohort study	56	54.00 ± 13.00	57.00	0	36.00	7.00	NA	PALS	20 ± 13.70months	ICM	2D-STE	Epsilon
Olsen	2024	Denmark	Prospective cohort study	956	74.00 ± 4.00	56.00	NA	91.00	30.00	NA	LASr, LAScd, LASct	31.50 ± 14.85months	ILR	2D-STE	EchoPAC (GE)
Pastore	2024	Italy	Prospective cohort study	310	67.50 ± 8.60	82.00	100	74.90	41.00	100.00	PALS	NA	ECG	2D-STE	TomTec/EchoPAC/Siemens
Pathan	2018	Australia	Prospective cohort study	538	65.00 ± 7.22	55.80	0	82.00	NA	12.00	LASr, LAScd, LASct	NA	ECG/Holter	2D-STE	TomTec
Pessoa-Amorim	2018	Portugal	Prospective cohort study	149	74.00 ± 8.60	51.00	100	88.50	37.80	43.60	PALS, PACS	7 ± 2.99days	ECG	2D-STE	Syngo VVI (Siemens)
Pu	2023	China	Prospective cohort study	372	48.26 ± 15.45	57.50	NA	23.40	7.00	0.00	LASr, LAScd	61.35 ± 21.58months	ECG/Holter/ICD	CMR feature-based tracking	Qmass (Medis)
Ramkumar	2019	Australia	Prospective cohort study	445	70.50 ± 4.20	45.00	NA	79.00	43.00	3.00	LASr	15.04 ± 3.68months	ECG/Holter	2D-STE	TomTec
Rasmussen	2019	Denmark	Retrospective cohort study	186	61.00 ± 13.00	62.00	NA	51.00	11.00	27.00	LASr	NA	ECG/Holter	2D-STE	EchoPAC (GE)
Saberniak	2023	Norway	Prospective cohort study	185	68.00 ± 13.00	67.00	NA	61.10	5.90	NA	LASr	852.51 ± 285.42days	ICM	2D-STE	EchoPAC (GE)
Saraiva	2020	Brazil	Prospective cohort study	192	53.00 ± 11.00	41.10	NA	27.30	5.10	0.80	LASr, LAScd, LASct	5.6 ± 2.7years	ECG/Holter/ICD	2D-STE	EchoPAC (GE)
Stassen	2022	Netherlands	Retrospective cohort study	125	50.00 ± 15.00	19.20	12	32.80	18.30	0.00	LASr	32.75 ± 11.74months	ECG/Holter	2D-STE	EchoPAC (GE)
Svartstein	2022	Denmark	Prospective cohort study	392	62.00 ± 11.50	77.00	100	30.40	8.90	100.00	LASr, LAScd, LASct	5.57 ± 0.82years	ICD-10	2D-STE	EchoPAC (GE)
Takagi	2023	Japan	Prospective cohort study	335	71.00 ± 16.00	43.00	7.8	74.00	15.00	21.00	PALS	18.5months	ECG	2D-STE	EchoPAC (GE)
Yafasov	2024	Denmark	Prospective cohort study	1,935	54.00 ± 17.00	43.00	NA	46.20	3.90	NA	LASr, LAScd, LASct	4.87 ± 0.89years	ICD-10	3DE	4D Auto LAQ (GE)
Zegkos	2021	Greece	Prospective cohort study	250	50.80 ± 15.80	67.20	NA	NA	NA	NA	LASr, LAScd	2.5 ± 1.2years	Holter/ICD	2D-STE	EchoPAC (GE)
Zheng	2023	Singapore	Prospective cohort study	157	61.00 ± 11.60		NA	NA	NA	NA	LAScd	3.5years	ILR	NA	NA
Ping	2025	China	Prospective cohort study	128	52.50 ± 9.60	63.30	60.1	33.60	13.30	20.30	LASr, LAScd, LASct	NA	ECG	3DE	4D auto LAQ (GE)
Shibata	2023	Japan	Prospective cohort study	100	60.10 ± 14.50	61.00	NA	46.00	17.00	2.00	LASr, LAScd	706.10 ± 335.19days	ECG	2D-STE	TomTec

ECG, electrocardiogram; ICD, International Classification of Diseases; ICM, International Classification of Diseases; ILR, implantable loop recorders; LA, left atrial; AF, atrial fibrillation; PALS, peak atrial longitudinal strain; LASr, left atrial reservoir strain; LAScd, left atrial conduit strain; LASct, left atrial systolic strain; PACS, peak atrial contraction strain; 2D-STE: two-dimensional speckle tracking echocardiography; 3D-STE: three-dimensional speckle tracking echocardiography.

### 3.2 Study quality

**Figure 2:**
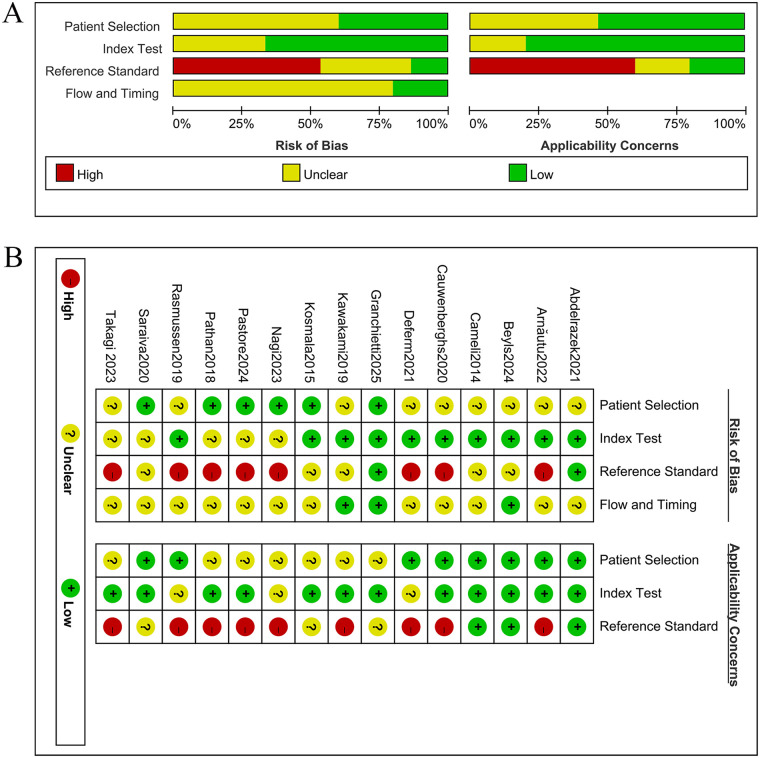
**(A)** Bar graph of risk of bias items and applicability; **(B)** summary of risk of bias and applicability.

QUADAS-2 results indicated a moderate overall risk of bias across the eligible studies (Figures 2A,B). The NOS scores for these studies ranged from 6 to 9, suggesting high quality. Detailed NOS scores are provided in Supplementary Tables S3, S4.

### 3.3 Meta-analysis results

#### 3.3.1 PALS and NOAF

**Figure 3:**
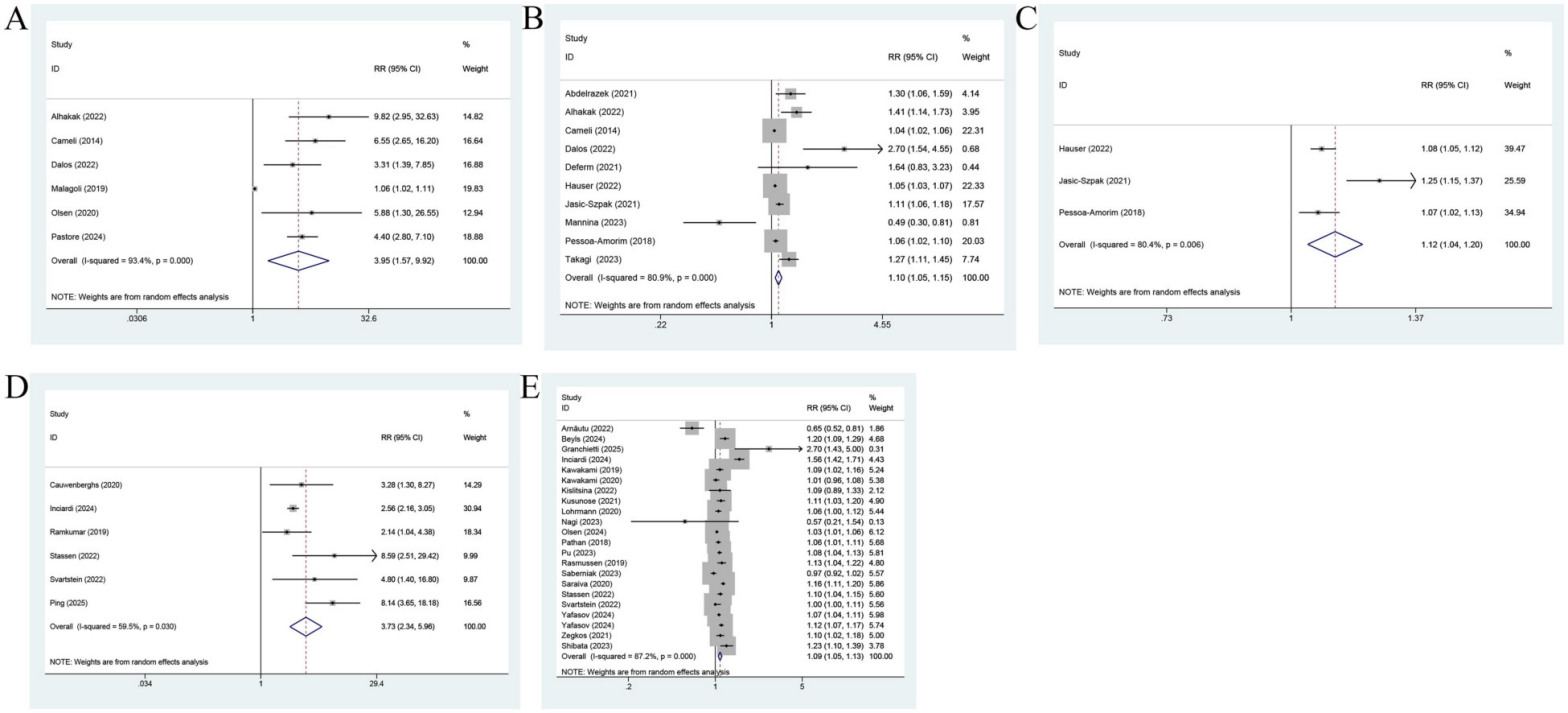
Forest plots for the correlation of PALS with NOAF as categorical **(A)** and continuous variables **(B)**, respectively; forest plot for the correlation of PACS with NOAF as a continuous variable **(C)**; forest plots for the correlation of LASr with NOAF as categorical **(D)** and continuous variables **(E)**, respectively.

Six studies (21–26) reported the correlation of PALS, as a dichotomous variable, with NOAF (Figure 3A). The meta-analysis revealed significant heterogeneity (*I*² = 93.4%, *P* = 0.001). The pooled results indicated an RR of 3.95 (95% CI = 1.57–9.92, *P* = 0.003), suggesting that low PALS was notably related to a higher incidence of NOAF. Subgroup analyses (Table 2) showed that lower PALS markedly predicted NOAF across different study types (*P* < 0.05 for all) and in the postoperative atrial fibrillation (POAF) cohort (*P* = 0.001). Meta-regression (Table 3) indicated that sample size, age, hypertension, and DM were not significant sources of heterogeneity. However, the proportion of males might be a contributing factor to the heterogeneity (*P* < 0.001).

**Table 2:** Subgroup analysis of LASPs and NOAF.

Subgroup analysis of PALS and NOAF								
Subgroup	PALS (dichotomous variables)				PALS (continuous variable)			
	Study	RR [95% CI]	*P* value	*I* ^2^	Study	RR [95%CI]	*P* value	*I* ^2^
**Total**	6	3.95[1.57–9.92]	0.003	93.40%	10	1.1 [1.05–1.15]	0.001	80.90%
Study design								
Case-control study	/	/	/	/	1	1.30 [1.06- 1.59]	0.011	NA
Retrospective cohort study	1	9.82 [2.95- 32.66]	0.001	NA	2	1.43 [1.17- 1.74]	0.001	0%
Prospective cohort study	5	3.35[1.29–8.71]	0.013	93.60%	7	1.08 [1.03- 1.12]	0.001	82.20%
Population								
POAF	3	4.47 [3.07- 6.49]	0.001	0%	4	1.09 [1.01- 1.18]	0.021	82.40%
non-POAF	3	3.58[0.68- 18.79]	0.132	89%	6	1.13 [1.03- 1.25]	0.014	82.70%
Region								
Africa	/	/	/	/	1	1.30 [1.06- 1.59]	0.011	NA
Europe	/	/	/	/	6	1.07 [1.03- 1.12]	0.001	79.70%
North America	/	/	/	/	2	0.88 [0.27- 2.86]	0.826	87.40%
Asia	/	/	/	/	1	1.27 [1.11- 1.45]	0.001	NA

Subgroup analysis of LASr and NOAF								
Subgroup	LASr (dichotomous variables)				LASr (continuous variable)			
	Study	RR [95% CI]	*P* value	*I* ^2^	Study	RR [95% CI]	*P* value	*I* ^2^
Total	6	3.73[2.34–5.96]	0.03	59.50%	22	1.09 [1.05–1.13]	0.001	87.20%
Study design								
Case-control study	/	/	/	/	1	1.09 [1.02–1.16]	0.009	NA
Retrospective cohort study	1	8.59 [2.51–29.42]	0.001	NA	6	1.03 [0.96–1.11]	0.395	80.70%
Prospective cohort study	5	3.35 [2.13–5.27]	0.001	55.60%	15	1.12 [1.07–1.17]	0.001	89.80%
Population								
POAF	1	4.80 [1.39–16.63]	0.013	NA	5	1.06 [0.95–1.17]	0.28	69.20%
non-POAF	5	3.67 [2.19–6.15]	0.001	65.40%	17	1.10 [1.06–1.14]	0.001	89.10%
Region								
Europe	3	4.68 [2.48–8.84]	0.001	0%	11	1.06 [1.02–1.11]	0.008	85.40%
North America	1	2.56 [2.15–3.04]	0.001	NA	3	1.22 [0.92–1.62]	0.17	95.90%
Oceania	1	2.14 [1.04–4.39]	0.038	NA	3	1.05 [1.01–1.10]	0.014	35.60%
Asia	1	8.14 [3.65–18.18]	0.001	NA	3	1.12 [1.05–1.19]	0.001	53.50%
Africa	/	/	/	/	1	0.57 [0.21–1.54]	0.269	NA
South America	/	/	/	/	1	1.16 [1.12–1.21]	0.001	NA

Subgroup analysis of LAScd and NOAF								
Subgroup	LAScd (continuous variable)							
	Study	RR [95% CI]	*P* value	*I* ^2^				
**Total**	16	1.09 [1.05–1.13]	0.001	81.6%,				
Study design								
Case-control study	/	/	/	/				
Retrospective cohort study	2	1.16 [1.12–1.21]	0.001	0%				
Prospective cohort study	14	1.07 [1.03–1.11]	0.001	77.30%				
Population								
POAF	2	1.00 [0.65–1.53]	0.993	29.20%				
non-POAF	14	1.10 [1.06–1.14]	0.001	81.20%				
Region								
Europe	8	1.04 [1.00–1.08]	0.055	71.20%				
North America	1	1.19 [1.08–1.31]	0.001	NA				
Oceania	1	1.16 [1.12–1.21]	0.001	NA				
Asia	4	1.12 [1.07–1.18]	0.001	21.70%				
Africa	1	1.67 [0.62–4.52]	0.313	NA				
South America	1	1.15 [1.09–1.22]	0.001	NA				

Subgroup analysis of LASct and NOAF								
Subgroup	LASct (continuous variable)							
	Study	RR [95% CI]	*P* value	*I* ^2^				
**Total**	21	1.13 [1.06–1.19]	0.001	89.90%				
Study design								
Case-control study	/	/	/	/				
Retrospective cohort study	5	1.13 [1.06–1.20]	0.001	46.40%				
Prospective cohort study	16	1.12 [1.05–1.20]	0.001	92.00%				
Population								
POAF	6	1.10 [1.06–1.15]	0.001	0%				
non-POAF	15	1.12 [1.05–1.21]	0.001	92.80%				
Region								
Europe	8	1.13 [1.08–1.17]	0.001	65.40%,				
North America	4	1.05[0.80–1.37]	0.725	92.90%,				
Multicentric	1	1.10 [1.02–1.19]	0.016	NA				
Africa	1	1.54 [0.56–4.20]	0.399	NA				
Oceania	3	1.14 [1.03–1.26]	0.001	78%				
South America	1	0.76 [0.71–0.82]	0.001	NA				
Asia	3	1.22 [1.04–1.44]	0.016	87.3%				

PALS, peak atrial longitudinal strain; NOAF, new-onset atrial fibrillation; RR, risk ratios; CI, confidence interval; POAF, postoperative atrial fibrillation; LASr, left atrial reservoir strain; LAScd, left atrial conduit strain; LASct, left atrial systolic strain.

**Table 3:** Regression analysis of LASPs and NOAF.

	Coefficient	95% CI	*P*
PALS (dichotomous)			
Sample size	−0.0006921	−0.0069032 to 0.0055191	0.827
Age	−0.0619607	−0.1834743 to 0.0595528	0.318
Male proportion	−0.0672607	−0.0907338 to −0.0437877	0.001
Hypertension	−0.0256232	−0.0673882 to 0.0161419	0.229
Diabetes mellitus	−0.0222443	−0.0869291 to 0.0424405	0.500
PALS (continuous)			
Sample size	−0.00000321	−0.0000124 to 0.00000596	0.492
Age	0.000074	−0.0019976 to 0.0021457	0.944
Male proportion	−0.0008508	−0.0018122 to 0.0001105	0.083
Hypertension	0.0004975	−0.0004718 to 0.0014668	0.314
Diabetes mellitus	0.0008876	−0.0002688 to 0.0020439	0.132
LASr (dichotomous)			
Sample size	−0.000114	−0.000211 to −0.000017	0.021
Age	−0.035523	−0.0591045 to −0.0119416	0.003
Male proportion	0.0174048	−0.0053688 to 0.0401784	0.134
Hypertension	−0.0072642	−0.0378419 to −0.0093666	0.001
Diabetes mellitus	−0.0303224	−0.0536484 to −0.0069965	0.011
LASr (continuous)			
Sample size	0.0000593	0.0000233 to 0.0000954	0.001
Age	0.0014047	−0.0052683 to 0.0080776	0.680
Male proportion	−0.0017137	−0.0053975 to 0.0019701	0.362
Hypertension	−0.0002297	−0.0034442 to 0.0029849	0.889
Diabetes mellitus	0.0019205	−0.0029869 to 0.0068278	0.443
LASct (continuous)			
Sample size	0.000089	0.0000322 to 0.0001459	0.002
Age	0.0020514	−0.0047544 to 0.0088572	0.555
Male proportion	0.0080468	0.0027631 to 0.0133306	0.003
Hypertension	0.0021318	−0.001669 to 0.0059325	0.272
Diabetes mellitus	0.0034418	−0.0030846 to 0.0099681	0.301
LAScd (continuous)			
Sample size	−0.00000494	−0.0000329 to 0.000023	0.729
Age	−0.000734	−0.0053636 to 0.0038955	0.756
Male proportion	−0.0001801	−0.0035353 to 0.0031752	0.916
Hypertension	0.0000477	−0.002015 to 0.0021103	0.964
Diabetes mellitus	0.0011438	−0.0025896 to 0.0048772	0.548

PALS, peak atrial longitudinal strain; LASr, left atrial reservoir strain; LAScd, left atrial conduit strain; LASct, left atrial systolic strain; PACS, peak atrial contraction strain.

Ten studies (21–23, 27–33) evaluated the link between PALS, as a continuous variable, and NOAF (Figure 3B). The meta-analysis detected evident heterogeneity (*I*² = 80.9%, *P* = 0.001). The outcomes displayed that for every one-unit decrease in PALS, the risk of NOAF increased by 1.1 times (95% CI = 1.05–1.15, *P* = 0.001). Subgroup analyses (Table 2) indicated that low PALS markedly predicted NOAF in African, European, and Asian populations (*P* < 0.05 for all), across different study types (*P* < 0.05 for all), and in both surgical and non-surgical populations (*P* < 0.05 for all). Meta-regression (Table 3) revealed that sample size, age, hypertension, DM, and the proportion of males were not significant sources of heterogeneity.

#### 3.3.2 PACS and NOAF

Three studies (29, 30, 32) examined the link between PACS, as a continuous variable, and NOAF (Figure 3C). The meta-analysis illustrated significant heterogeneity (*I*² = 80.4%, *P* = 0.006). The findings indicated that low PACS was linked to a 1.12-fold elevated risk of NOAF relative to high PACS (95% CI = 1.04–1.20, *P* = 0.002).

#### 3.3.3 LASr and NOAF

Six studies (34–39) assessed the relationship between LASr, as a dichotomous variable, and NOAF (Figure 3D). The meta-analysis uncovered significant heterogeneity (*I*² = 59.5%, *P* = 0.03). The pooled results yielded an RR of 3.73 (95% CI = 2.34–5.96, *P* = 0.001), indicating that low LASr was notably linked to a higher incidence of AF. Subgroup analyses (Table 2) revealed that low LASr significantly predicted NOAF across different study types (*P* < 0.05 for all), in both surgical and non-surgical populations (*P* < 0.05 for all), and across different geographical regions (*P* < 0.05 for all). Meta-regression (Table 3) indicated that the proportion of males was not a source of heterogeneity. Nonetheless, sample size, age, hypertension, and DM might be contributing factors to significant heterogeneity (*P* < 0.05).

Twenty-two studies (34–36, 40–57) investigated the correlation of LASr, as a continuous variable, with AF incidence (Figure 3E). The meta-analysis revealed evident heterogeneity (*I*² = 87.2%, *P* = 0.001). The results indicated that low LASr was related to a 1.09-fold increased risk of NOAF relative to high LASr (95% CI = 1.05–1.13, *P* = 0.001). Subgroup analyses (Table 2) displayed significant associations of LASr with NOAF in the non-POAF cohort (*P* = 0.001), prospective cohort and case-control studies (*P* < 0.05 for all), and in European, Oceanian, Asian, and South American populations (*P* < 0.05 for all). No significant link was observed in the POAF cohort, retrospective cohort studies, or in North American and African populations. Meta-regression (Table 3) illustrated that age, proportion of males, hypertension, and DM were not significant sources of heterogeneity. Sample size, however, might be a contributing factor to heterogeneity (*P* = 0.001).

#### 3.3.4 LAScd and NOAF

**Figure 4:**
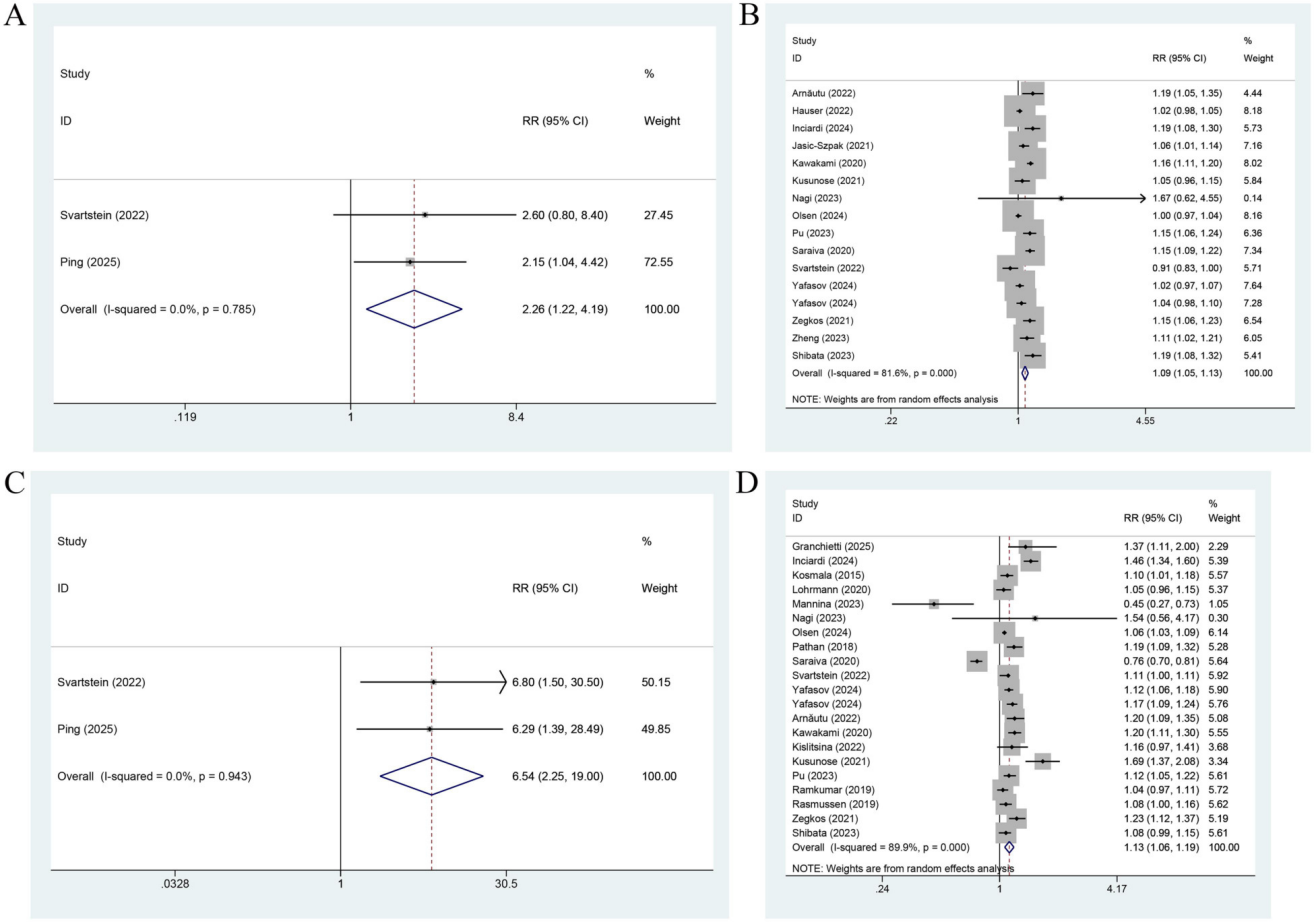
Forest plots for the correlation of LAScd with NOAF as categorical **(A)** and continuous variables **(B)**, respectively; forest plots for the correlation of LASct with NOAF as categorical **(C)** and continuous variables **(D)**, respectively.

Two studies (36, 39) examined the correlation of LAScd, as a dichotomous variable, with NOAF (Figure 4A). The meta-analysis found no significant heterogeneity (*I*² = 0%, *P* = 0.785). The pooled result was RR = 2.26 (95% CI = 1.22–4.19, *P* = 0.009), indicating that low LAScd was notably related to a higher incidence of AF.

Sixteen studies (29, 30, 34, 36, 40, 44, 46, 48, 49, 51, 54–58) evaluated the correlation of LAScd, as a continuous variable, with NOAF (Figure 4B). The meta-analysis revealed significant heterogeneity (*I*² = 81.6%, *P* = 0.001). The findings indicated that low LAScd was related to a 1.09-fold elevated risk of NOAF relative to high LAScd (95% CI = 1.05–1.13, *P* = 0.001). Subgroup analyses (Table 2) showed marked associations of LAScd with NOAF in the non-POAF cohort (*P* = 0.001), across different study types (*P* < 0.05 for all), and in North American, Oceanian, Asian, and South American populations (*P* < 0.05 for all). No significant link was identified in the POAF cohort or in European and African populations. Meta-regression (Table 3) showed that sample size, age, proportion of males, hypertension, and DM were not significant sources of heterogeneity.

#### 3.3.5 LASct and NOAF

Two studies (36, 39) analyzed the correlation of LASct, as a dichotomous variable, and NOAF (Figure 4C). The meta-analysis found no significant heterogeneity (*I*² = 0%, *P* = 0.943). The pooled result was RR = 6.54 (95% CI = 2.25–19.0, *P* = 0.001), suggesting that low LASct was notably correlated with a higher incidence of AF. Twenty-one studies (31, 34, 36, 38, 40, 42, 44–52, 54–57, 59) examined the relationship between LASct, as a continuous variable, and NOAF (Figure 4D). The meta-analysis revealed significant heterogeneity (*I*² = 89.9%, *P* = 0.001). Low LASct was associated with a 1.13-fold higher risk of NOAF compared with high LASct (95% CI: 1.06–1.19, *P* = 0.001). Subgroup analyses (Table 2) showed that this association remained significant across subgroups defined by surgical status, study type, and geographic region (Europe, multicenter studies, Oceania, South America, and Asia; all *P* < 0.05), whereas no significant link was observed in North America and Africa. Meta-regression analyses (Table 3) indicated that age, hypertension, and DM were not significant sources of heterogeneity, whereas sample size and proportion of male participants may contribute to the observed heterogeneity (*P* < 0.05).

### 3.4 Sensitivity analysis

**Figure 5:**
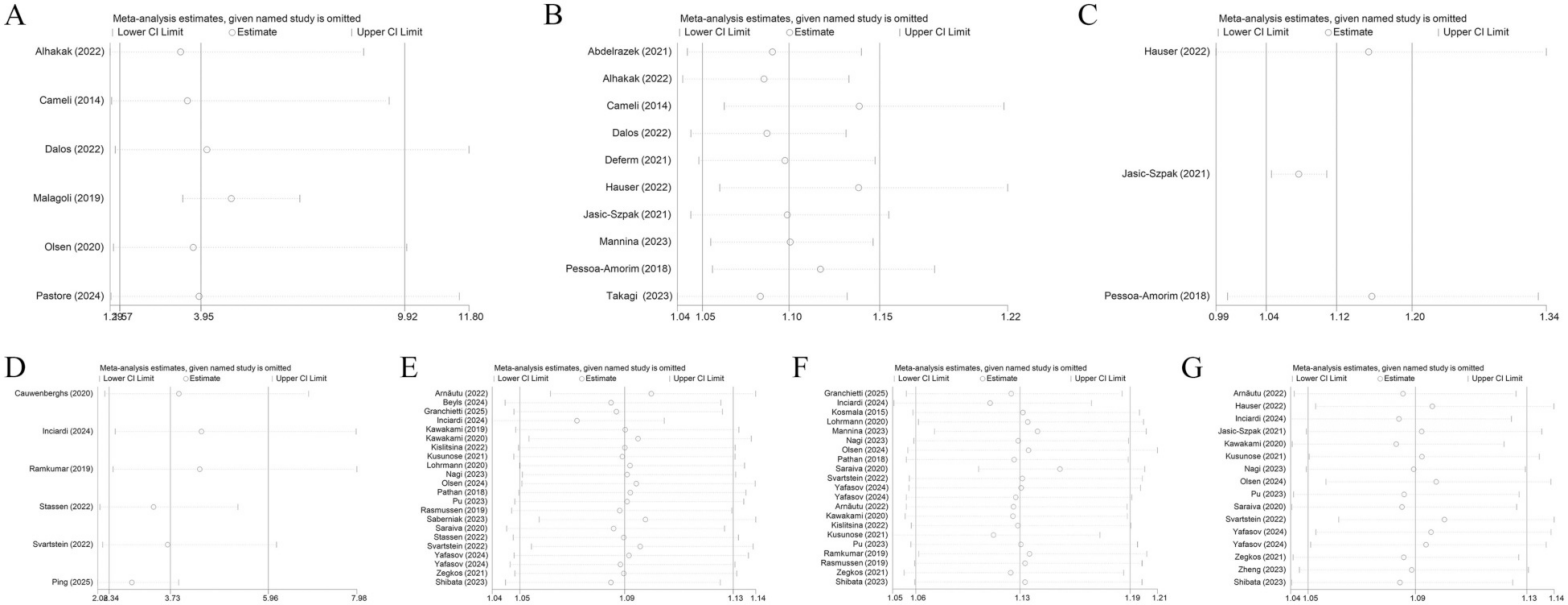
Sensitivity analysis of PALS and NOAF as categorical **(A)** and continuous variables **(B)**, respectively; sensitivity analysis of PACS and NOAF as a continuous variable **(C)**; sensitivity analysis of LASr and NOAF as categorical **(D)** and continuous variables **(E)**, respectively; sensitivity analysis of LASct and NOAF as a continuous variable **(F)**; sensitivity analysis of LAScd and NOAF as a continuous variable **(G)**.

Sensitivity analyses revealed that the results of the current meta-analysis were stable and reliable (Figures 5A–G). The findings for PALS, LASr, LAScd, and LASct were not notably influenced by any single eligible study. Due to the limited number of studies reporting LAScd and LASct as dichotomous variables, corresponding sensitivity analyses were not implemented.

### 3.5 Publication bias

Egger's test and funnel plots were employed to evaluate publication bias. As illustrated in Supplementary Figure S1A–F, the funnel plot for PALS as a dichotomous variable exhibited asymmetry, while the plots for other metrics were symmetrical. Egger's test results were PALS-dichotomous (*P* = 0.007), PALS-continuous (*P* = 0.067), LASr-dichotomous (*P* = 0.123), LASr-continuous (*P* = 0.297), LAScd-continuous (*P* = 0.168), and LASct-continuous (*P* = 0.342). Due to the presence of publication bias for PALS as a dichotomous variable, the trim-and-fill method was applied, yielding a statistically significant result (*P* = 0.004). This indicated the robustness of the study findings.

### 3.6 Meta-analysis of diagnostic tests

#### 3.6.1 Threshold effects

Data were imported into Meta-Disc for analysis. Threshold effect testing for PALS, LASr, and LASct yielded Spearman correlation coefficients between the logarithm of sensitivity and the logarithm of (1-specificity) of 0 (*P* = 1), 0.5 (*P* = 0.207), and 0.657 (*P* = 0.156), respectively. These outcomes indicated the absence of a threshold effect.

#### 3.6.2 Pooled effect sizes for diagnostic tests

**Figure 6:**
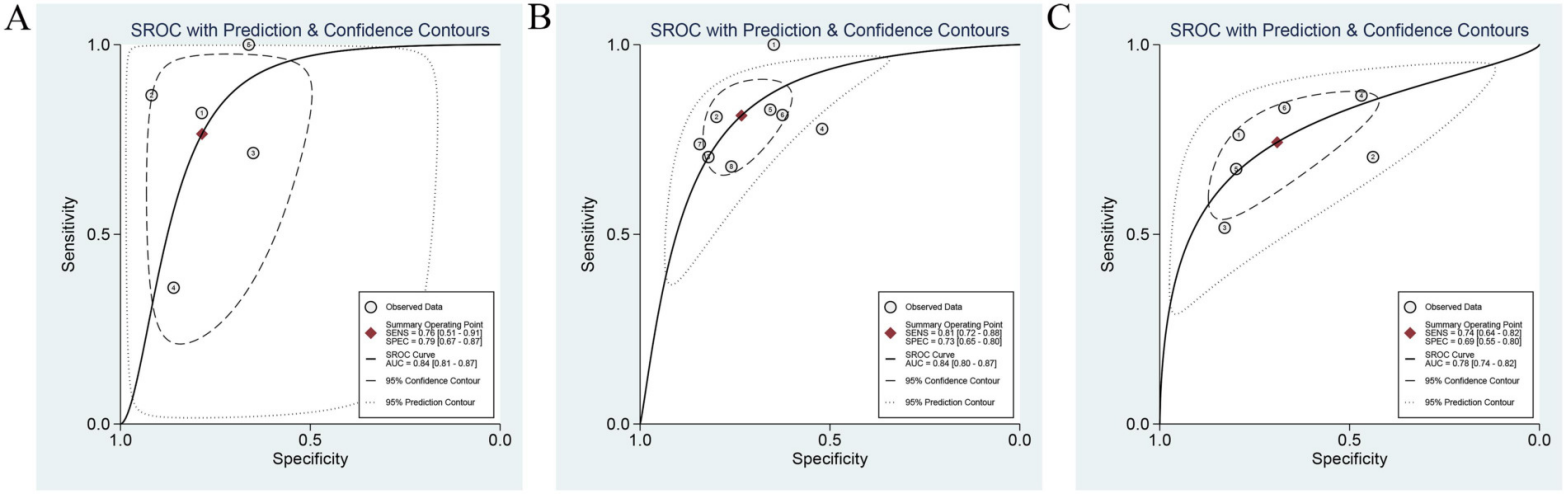
Summary receiver operating characteristic (sROC) curves for PALS **(A)**, LASr **(B)**, and LASct **(C)** in predicting NOAF.

Based on heterogeneity evaluation, a bivariate random-effects model was selected. For PALS, the pooled sensitivity, specificity, negative likelihood ratio, positive likelihood ratio, and diagnostic odds ratio were 0.76 (95% CI: 0.51–0.91), 0.79 (0.67–0.87), 3.56 (2.24–5.65), 0.30 (0.13–0.69), and 11.88 (3.94–35.85), respectively. For LASr, the pooled sensitivity, specificity, negative likelihood ratio, positive likelihood ratio, and diagnostic odds ratio were 0.81 (0.72–0.88), 0.73 (0.65–0.80), 3.05 (2.38–3.89), 0.25 (0.17–0.37), and 11.95 (7.39–19.30), respectively. For LASct, the pooled sensitivity, specificity, negative likelihood ratio, positive likelihood ratio, and diagnostic odds ratio were 0.74 (0.64–0.82), 0.69 (0.55–0.80), 2.40 (1.73–3.32), 0.37 (0.29–0.48), and 6.44 (4.34–9.56), respectively. These findings are detailed in Supplementary Figures S2–S4. The SROC curves, with true positive rate as the *y*-axis and false positive rate as the *x*-axis, are presented in Figures 6A–C. The pooled AUCs for PALS, LASr, and LASct were 0.84, 0.84, and 0.78, respectively, indicating good diagnostic value for these 3 parameters.

#### 3.6.3 Clinical utility

**Figure 7:**
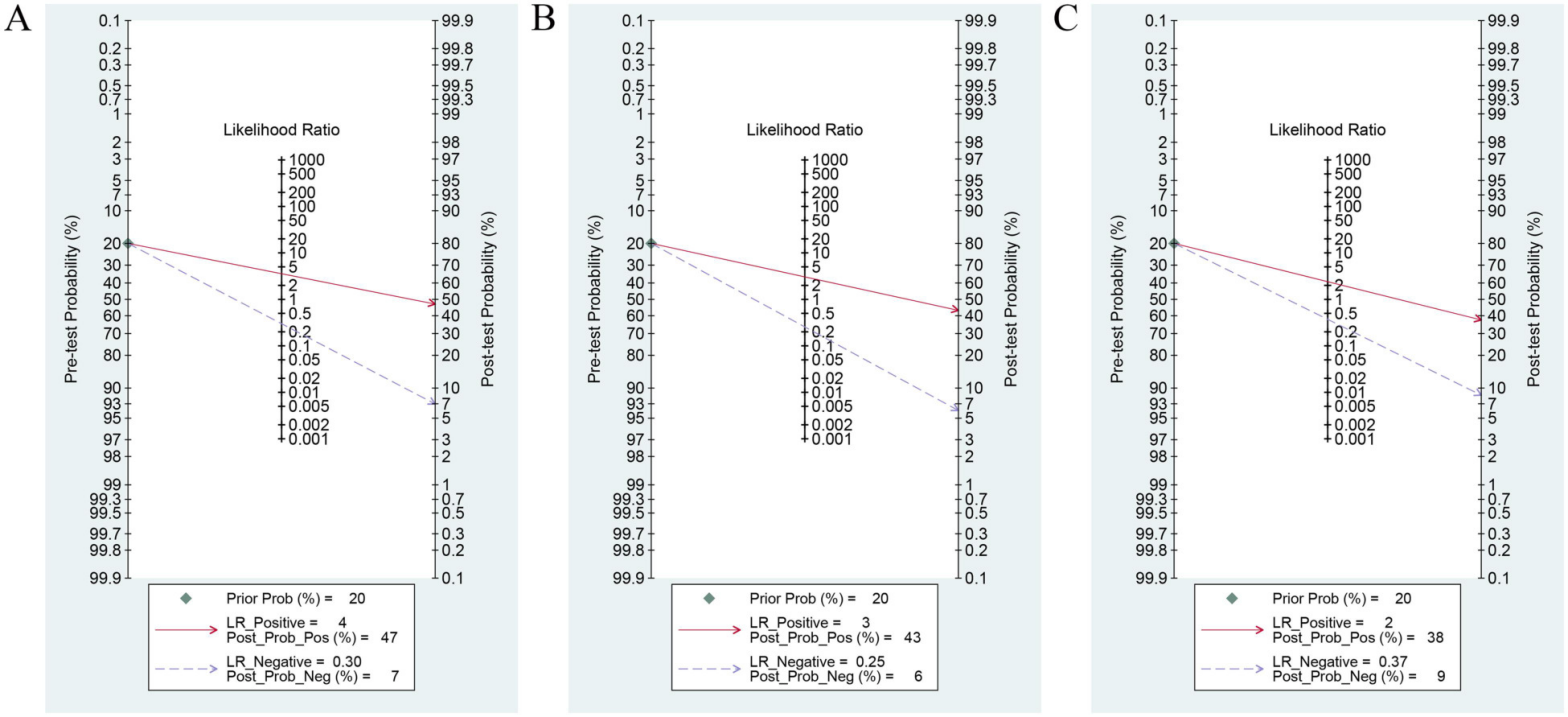
Fagan nomograms illustrating the clinical utility of PALS **(A)**, LASr **(B)**, and LASct **(C)** for predicting NOAF.

In the Fagan nomogram analysis, a pre-test probability of NOAF of 20% was established. This is based on the premise that the majority of subjects in this meta-analysis were at intermediate to high risk due to underlying cardiovascular conditions, projecting a higher anticipated risk of NOAF than in the general population. This probability aids in a more clinically relevant evaluation of the predictive value of LASPs. Analysis using Fagan nomograms to evaluate the clinical utility of PALS, LASr, and LASct in predicting NOAF (Figures 7A–C) revealed that with a pre-test probability of 20%, the post-test probabilities increased to 49%, 43%, and 38% for PALS, LASr, and LASct, respectively. This indicated that these 3 LASPs possess considerable diagnostic value in clinical application.

#### 3.6.4 Deeks' publication bias test

**Figure 8:**
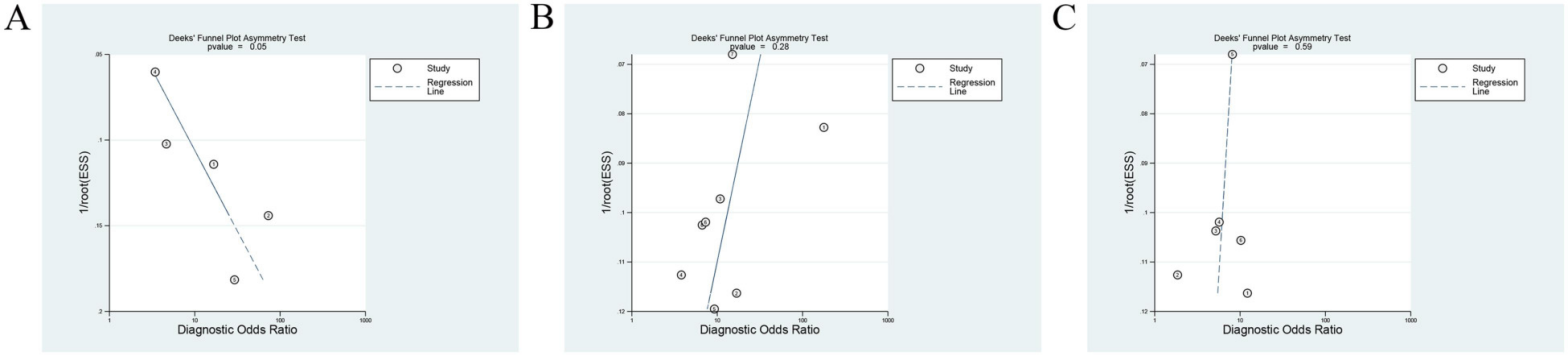
Funnel plots for publication bias assessment of PALS **(A)**, LASr **(B)**, and LASct **(C).**

Stata 15.0 was utilized for publication bias testing. As depicted in Figures 8A–C, the funnel plots for PALS, LASr, and LASct exhibited symmetry (*P* > 0.05 for all), suggesting no significant publication bias.

## 4 Discussion

This meta-analysis comprehensively evaluated the prognostic value of left atrial strain in subjects with NOAF. Thirty-nine studies, encompassing 20,681 individuals, were incorporated. Our findings demonstrated that reduced LASPs hold significant diagnostic value for NOAF. Specifically, PALS, PACS, LASr, LAScd, and LASct were all significantly linked to NOAF. Furthermore, the diagnostic meta-analysis of PALS, LASr, and LASct in predicting the risk of NOAF revealed pooled AUCs of 0.84, 0.84, and 0.78, respectively. This signified good diagnostic value for these 3 LASPs. Collectively, the findings confirm the important role of several LASPs in predicting NOAF.

Notably, the meta-analysis fully incorporating data on the left atrial contractile strain (LASct, i.e., left atrial pump strain) revealed that LASct analyzed as a continuous variable was significantly associated with NOAF (*P* = 0.001, RR = 1.13, 95% CI: 1.06–1.19). This finding suggests that impaired left atrial active contractile function may be more stably linked to the development of AF, rather than manifesting solely as a sharply increased risk below a specific cutoff. Nevertheless, the analysis of LASct as a continuous variable still exhibited high heterogeneity (*I*² = 89.9%). This indicated that the magnitude of the effect of LASct may be influenced by differences in the composition of participants, clinical settings, techniques of measurement, and strategies for ascertainment of endpoints. Therefore, current evidence supports LASct as a functional parameter with potential predictive value, although its optimal application requires further validation in large-scale, prospective studies using standardized methodologies. Undeniably, prior research has been published on left atrial strain and the risk of NOAF (60). However, that literature included only 7 studies published between 2019 and 2024, involving 6,349 subjects. Additionally, it solely considered PALS as the LASP for diagnosing the risk of NOAF. The current meta-analysis incorporated a substantial number of high-quality studies published between 2024 and 2025 and encompassed commonly used parameters for diagnosing NOAF, such as PACS, LASr, LAScd, and LASct. Furthermore, our research included an assessment of diagnostic performance and clinical utility. To our knowledge, this represents the most recent and comprehensive meta-analysis synthesizing the correlation of left atrial strain with the risk of NOAF.

Numerous meta-analyses have found that left atrial strain can also be utilized in the diagnosis of other cardiovascular and cerebrovascular diseases. For instance, a meta-analysis by Lacy et al., involving 2,660 subjects, demonstrated reduced PALS in symptomatic aortic stenosis (AS) individuals with cardiovascular events relative to those without symptoms or cardiovascular events. This indicates PALS as an independent predictor of cardiovascular events in AS individuals (61). Jia et al. (62) found in a meta-analysis of 17 investigations that PALS is an independent predictor of all-cause mortality and cardiac hospitalization in individuals with heart failure. Guo et al. (63) reported in a meta-analysis that LASr can independently predict the risk of ischemic stroke. Our analysis, which found LASPs to be predictive of NOAF risk, aligns broadly with findings from studies on cardiovascular and cerebrovascular diseases.

While the precise mechanisms underlying the correlation of LASPs with NOAF remain not fully elucidated, they can be explained from several perspectives. Firstly, reservoir strain (PALS/LASr) is a composite representation of left atrial compliance, elastic energy storage, and left ventricular basal recoil. It exhibits high sensitivity to left ventricular diastolic pressure, pericardial restriction, mitral regurgitation, and heart rate loading (64, 65). Specifically, PALS, anchored by the P wave, is more sensitive to low voltage zone expansion, signal fractionation, and localized conduction delay, thereby identifying potential critical thresholds even before significant atrial volume enlargement (66). In contrast, LASr primarily reflects reservoir phase compliance and elastic recoil. It is influenced by atrial wall fibrosis and constrained by left ventricular traction and diastolic loading. Reduced LASr is related to elevated left atrial pressure and fibrosis, serving as a potential marker for AF onset, progression, and recurrence (67). Conduit strain (LAScd) predominantly reflects passive filling driven by the atrio-ventricular pressure gradient during early diastole and left atrial elastic recoil (68). It serves as a crucial supplement for assessing left atrio-ventricular coupling and early diastolic function. Influenced by left ventricular relaxation, atrial pressure waveforms, and pulmonary vein compliance, conduit strain has stronger implications for AF susceptibility in individuals with heart failure and high filling pressures, mitral valve disease, or elevated pulmonary circulation load (56, 69). Systolic strain (LASct) reflects the active pumping function of the atrium under sinus rhythm. It is primarily affected by myofilament calcium coupling and electro-mechanical phase desynchronization (70). When atrial conduction heterogeneity and myocardial structural remodeling coexist, LASct typically declines earlier. Previous studies have indicated that impaired baseline strain is linked to higher AF recurrence rates and less reverse remodeling (71). Reduced PACS is more likely to represent insufficient contractile muscle mass or myofilament dysfunction (13, 72), correlating with atrial fibrosis, inflammatory infiltration, and ischemic injury (73). This aligns with the stronger effect observed in POAF (74) and individuals with structural heart disease (75). Collectively, these findings underscore the importance of LASPs in predicting NOAF, particularly before significant atrial volume enlargement occurs.

The current meta-analysis suggests that, in clinical practice, this could help identify high-risk individuals who would benefit from closer monitoring, more frequent follow-ups, or early supportive interventions. The findings support including LASPs in clinical AF risk stratification tools, particularly for individuals without electrocardiogram abnormalities but with underlying atrial dysfunction. This enables early intervention and personalized management.

Our meta-analysis has several limitations that warrant consideration. Firstly, many eligible studies were retrospective. Additionally, participants in these studies came from various countries and regions. Not all studies were conducted in multicenter settings; many were single-center studies. These factors may introduce unavoidable selection bias and confounding influences. Secondly, considerable heterogeneity existed in the measurement methods for left atrial strain across studies, including variations in ultrasound equipment, analysis software, and inconsistent definitions of regions of interest and boundaries, which may affect the accuracy of absolute strain values and effect size estimates. Thirdly, low to high degrees of heterogeneity were observed among the included studies. Certain subgroup analyses were based on small sample sizes or inconsistent follow-up durations. This may be linked to the observed heterogeneity and potential publication bias. Although the meta-regression identified partial sources of heterogeneity, the results should still be interpreted with caution. Additionally, further subgroup analyses were not possible due to the limited number of studies per technical configuration and the diversity of measurement approaches, which may also contribute to the observed heterogeneity. Further prospective, high-quality, and multicenter clinical trials are needed to corroborate these findings.

## 5 Conclusion

This meta-analysis highlights the substantial diagnostic value of LASPs in predicting NOAF, establishing them as a promising non-invasive marker for evaluating NOAF and informing clinical decisions. However, given the limitations of this investigation, including a preponderance of retrospective research, considerable heterogeneity, and potential selection bias, larger-scale, multicenter, prospective clinical studies are necessary to validate LASPs as routine clinical tools for managing NOAF and aiding in risk stratification and treatment decisions.

## Data Availability

The original contributions presented in the study are included in the article/Supplementary Material, further inquiries can be directed to the corresponding author.
